# Rats deficient C-type natriuretic peptide suffer from impaired skeletal growth without early death

**DOI:** 10.1371/journal.pone.0194812

**Published:** 2018-03-22

**Authors:** Toshihito Fujii, Keisho Hirota, Akihiro Yasoda, Akiko Takizawa, Naomi Morozumi, Ryuichi Nakamura, Takafumi Yotsumoto, Eri Kondo, Yui Yamashita, Yoriko Sakane, Yugo Kanai, Yohei Ueda, Ichiro Yamauchi, Shigeki Yamanaka, Kazumasa Nakao, Koichiro Kuwahara, Toshimasa Jindo, Mayumi Furuya, Tomoji Mashimo, Nobuya Inagaki, Tadao Serikawa, Kazuwa Nakao

**Affiliations:** 1 Department of Diabetes, Endocrinology and Nutrition, Kyoto University Graduate School of Medicine, Kyoto, Japan; 2 Department of Physiology, Medical College of Wisconsin, Milwaukee, Wisconsin, United States of America; 3 Asubio Pharma Co., Ltd., Kobe, Japan; 4 Department of Maxillofacial Surgery, Kyoto University Graduate School of Medicine, Kyoto, Japan; 5 Department of Cardiovascular Medicine, Shinshu University Graduate School of Medicine, Matsumoto, Japan; 6 Medical Innovation Center, Kyoto University Graduate School of Medicine, Kyoto, Japan; 7 Genome Editing Research and Development (R&D) Center and Institute of Experimental Animal Sciences, Graduate School of Medicine, Osaka University, Osaka, Japan; 8 Laboratory of Pharmacology, Osaka University of Pharmaceutical Sciences, Takatsuki, Japan; Kyungpook National University School of Medicine, REPUBLIC OF KOREA

## Abstract

We have previously investigated the physiological role of C-type natriuretic peptide (CNP) on endochondral bone growth, mainly with mutant mouse models deficient in CNP, and reported that CNP is indispensable for physiological endochondral bone growth in mice. However, the survival rate of CNP knockout (KO) mice fell to as low as about 70% until 10 weeks after birth, and we could not sufficiently analyze the phenotype at the adult stage. Herein, we generated CNP KO rats by using zinc-finger nuclease-mediated genome editing technology. We established two lines of mutant rats completely deficient in CNP (CNP KO rats) that exhibited a phenotype identical to that observed in mice deficient in CNP, namely, a short stature with severely impaired endochondral bone growth. Histological analysis revealed that the width of the growth plate, especially that of the hypertrophic chondrocyte layer, was markedly lower and the proliferation of growth plate chondrocytes tended to be reduced in CNP KO rats. Notably, CNP KO rats did not have malocclusions and survived for over one year after birth. At 33 weeks of age, CNP KO rats persisted significantly shorter than wild-type rats, with closed growth plates of the femur in all samples, which were not observed in wild-type rats. Histologically, CNP deficiency affected only bones among all body tissues studied. Thus, CNP KO rats survive over one year, and exhibit a deficit in endochondral bone growth and growth retardation throughout life.

## Introduction

C-type natriuretic peptide (CNP) is a member of the natriuretic peptide family along with atrial natriuretic peptide (ANP) and brain natriuretic peptide (BNP) [1]. ANP and BNP are hormones mainly produced from the atrium and ventricle of the heart, respectively. These peptides act by binding a membrane-bound guanylyl cyclase receptor, natriuretic peptide receptor (NPR)-A, in the heart, kidney, and blood vessels [2]. In contrast, CNP is ubiquitously distributed but acts as a local regulator by binding another membrane-bound guanylyl cyclase receptor, NPR-B, in a wide variety of tissues, including the brain, gonads, and bone [2]. We have determined that CNP/NPR-B signaling is crucial for endochondral bone growth using knockout (KO) mouse models. Systemic and cartilage-specific CNP or NPR-B KO mice both exhibit a prominent short stature phenotype owing to impaired skeletal growth [3–7]. In contrast, transgenic mice with cartilage-specific overexpression of CNP or increased circulating levels of CNP show a skeletal overgrowth phenotype [8–11]. In humans, not only homozygous loss-of-function mutations in the NPR-B gene, which are the causes of acromesomelic dysplasia type Maroteaux, a form of skeletal dysplasia with severely impaired endochondral bone growth [12, 13], but also heterozygous mutations in that gene [14–18], reportedly caused impaired skeletal growth. Furthermore, a recent study showed that heterozygous loss-of-function mutations in the CNP gene also cause a short stature in humans [19]. Conversely, both gain-of-function mutations in the gene encoding NPR-B and increased CNP expression by balanced chromosomal translocations reportedly caused skeletal-overgrowth in humans [20–25]. These facts indicate that CNP/NPR-B signaling is a pivotal stimulator of endochondral bone growth in humans as well as in mice.

It is often stated that there are limitations to the use of mouse models in some research areas and rat models are superior for investigating human physiology because of their greater physiological similarity to humans [26]. With regard to CNP, around 30% of both systemic and cartilage-specific CNP KO mice die before adulthood, possibly due to malocclusion and malnutrition [3, 7], and we could not evaluate the phenotypes of CNP KO mice over a long term. To investigate further the physiological roles of CNP/NPR-B signaling *in vivo*, we determined to investigate those roles using other animal species and aimed to establish CNP KO rat models. However, the inability to utilize germ line-competent rat embryonic stem cells has made it difficult to develop KO rat lines. Recently, a novel gene-targeting technology, which employs zinc-finger nucleases (ZFNs), has been used to develop KO rat lines [27]. ZFNs are engineered zinc-finger proteins containing nucleotide sequence-specific DNA binding domains with the endonuclease activity of the restriction enzyme FokI. The endonuclease creates site-specific, double-stranded DNA breaks, which can lead to the imperfect repair of such double-stranded breaks by the non-homologous end-joining pathway. Such imperfect repair results in a high frequency of mutations and functional knockouts of targeted genes. In this study, we generated CNP KO rats using ZFN-mediated genome editing and analyzed their phenotypes.

## Materials and methods

### Animals

Animal experiments on CNP KO rats and wild-type (WT) rats were performed using F344/Stm rats deposited with the National Bio Resource Project for Rats in Japan (www.anim.med.kyoto-u.ac.jp/nbr). All animal care and experiments were conducted under the Guidelines for Animal Experiments of Kyoto University and Asubio Pharma Co., Ltd., and approved by the Animal Research Committee of Kyoto University (Permit number: MedKyo07598) and by the committee for ethics in animal experiments of Asubio Pharma Co., Ltd.

### Generation of CNP KO rats by ZFN-mediated genome editing technology

**Fig 1:**
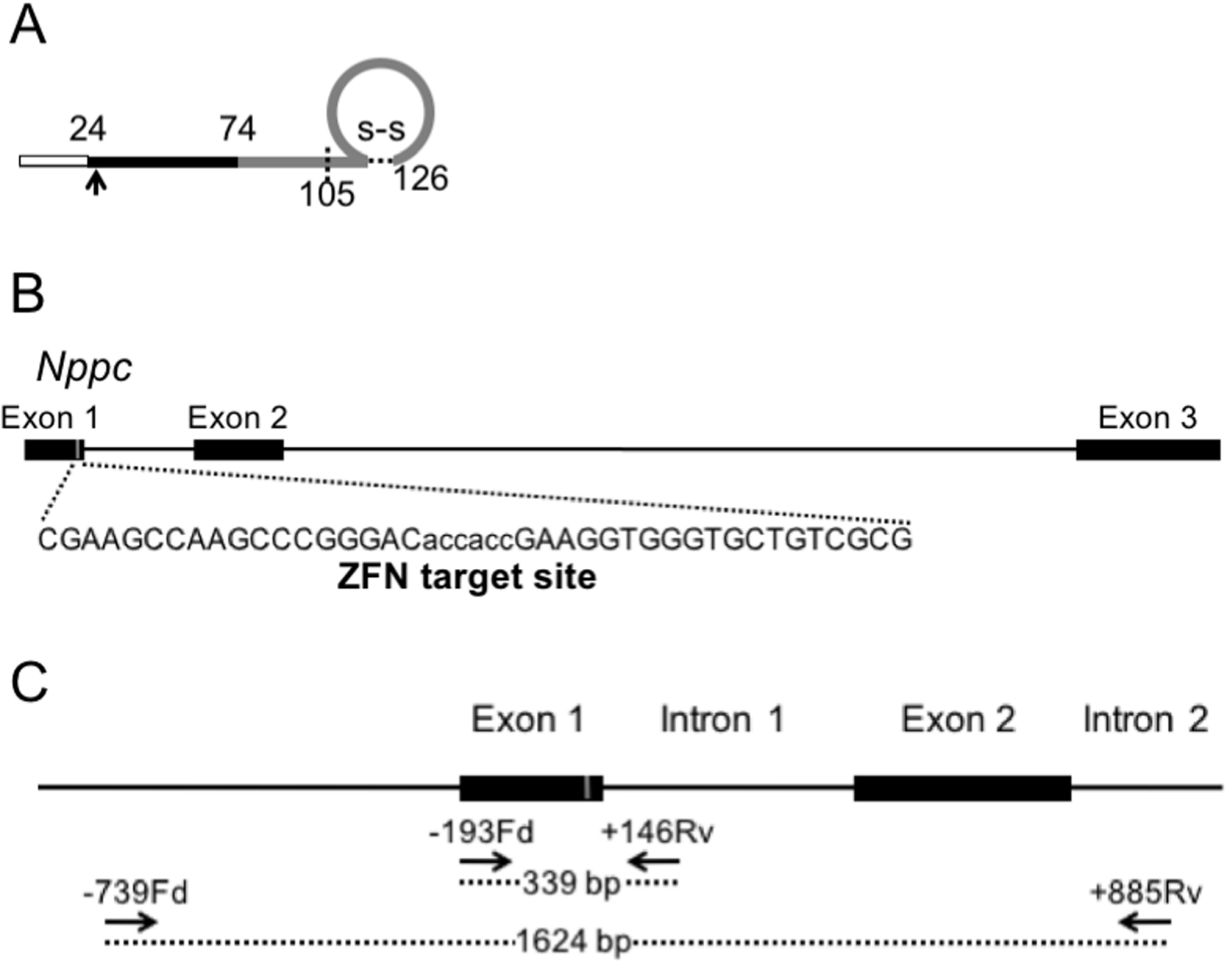
Generation of targeted mutations in the CNP-encoding gene *Nppc* by ZFN. (A) Schematic representation of the CNP protein and the target site within it (arrow). The empty (white) portion represents the signal peptide (23 a.a.), the filled (black) portion represents the NT-proCNP (50 a.a.), and the gray portion indicates the mature CNP with either 53 or 22 a.a. (B) Schematic representation of the target site in the rat *Nppc* (NC_005108.4) locus. (C) Schematic representation of the locations of PCR primers for genotyping.

Custom-designed ZFN plasmids for targeting the gene that encodes rat CNP (*Nppc*) were obtained from Sigma-Aldrich. The design, cloning, and validation of the ZFNs were performed by Sigma-Aldrich, and practical methods are described in detail [28, 29]. Selected ZFNs were designed to target exon 1 of rat *Nppc*, which encodes the N-terminus of proCNP (target sequence: CGAAGCCAAGCCCGGGACaccaccGAAGGTGGGTGCTGTCGCG; nucleotides 179–221, NC_005108.4) (Fig 1A and 1B). Microinjection of ZFN-encoding mRNA into rat pronuclear stage zygotes was performed before the embryos were transferred into the oviducts of pseudopregnant rats. The resulting offspring were screened for ZFN-induced mutations at the *Nppc* target site. PCR amplification from genomic DNA isolated from tail biopsies was performed as previously reported using two primer sets designed to amplify small (339-bp) and large (1624-bp) fragments as shown in Fig 1C and in Table 1. PCR products were sequenced using BigDye terminator v3.1 cycle sequencing mix and the standard protocol for an Applied Biosystems 3130 DNA Sequencer (Carlsbad, CA, USA). Analysis of off-target sites was performed as detailed in our previous manuscripts [28, 29].

**Table 1:** Sequences of the genotyping PCR primers for the detection of mutations in the CNP gene.

Forward	Primer sequence	Reverse	Primer sequence	Amplicon
-739Fd	5'-GGGATCTCAACCCACTTCCT-3'	+885Rv	5'-AAATCCAGACATTTGCCTGC-3'	1624-bp
-193Fd	5'-CACAGCAGTAGGACCCGTG-3'	+146Rv	5'-ACTTCTAGGGTTCGTTCCCC-3'	339-bp

### Quantitative RT-PCR

Total RNA was extracted from the brains of 10-week-old rats using Isogen reagent (Nippon Gene, Tokyo, Japan). Quantitative RT-PCR was subsequently performed using a TaqMan^®^ RT-PCR assay with primers specific for *Nppc* obtained from the manufacturer (Applied Biosystems).

### Measurement of plasma NT-proCNP concentrations

Plasma levels of N-terminal proCNP (NT-proCNP), as the representative for CNP production, in 10-week-old rats were measured using a proCNP, N-terminal, ELISA Kit (BI-20872, Biomedica Medizinprodukte GmbH & Co KG, Wien, Austria), according to the manufacturer’s protocol.

### Body weight and body length

Body weight and body length, measured as the naso-anal length, of CNP KO and WT rats were measured every week from two to ten weeks of age. We also measured them at the autopsy of rats at 33 weeks of age. The naso-anal length was measured by a ruler under anesthesia with an IP injection of 30 mg/kg pentobarbital (Sigma-Aldrich Japan, Tokyo) or inhalation of isoflurane (Pfizer, NY, USA).

### Skeletal analysis

Rats were subjected to soft X-ray analysis (30 kVp, 5 mA for one minute; Softron Type SRO-M5, Softron, Tokyo, Japan). Bone lengths were measured on soft X-ray film.

### Measurement of bone mineral density

Three female CNP KO rats and 6 female WT rats at 8 weeks of age were used for the analysis of bone mineral density. Isolated femurs of rats were subjected to computerized tomography (CT) scanning (Latheta LCT-200; Hitachi Aloka Medical, Tokyo, Japan). Parameters for the CT scans were as follows: tube voltage, 50 kVp; tube current, 0.5 mA; integration time, 3.6 ms; axial field of view, 48 mm, with an isotropic voxel size of 48 μm.

### Histological analysis of growth plates

For light microscopy, sections of the tibial growth plates were made from paraffin-embedded specimens and stained with Alcian blue-hematoxylin and eosin (H&E) as previously described [11]. For immunohistochemical detection of type II and type X collagens, tissue sections were incubated with either anti-type II collagen antibody (1320–01, Southern Biotech, AL, USA) or anti-type X collagen antibody (LB-0092, LSL, Co., Ltd., Japan), and immunostaining was performed as described previously [11].

### Bromodeoxyuridine (BrdU) analysis of the growth plate

BrdU (05650, Nacalai Tesuque, Kyoto, Japan) was IP injected into three-week-old CNP KO rats or WT rats at a dose of 50 μg/g body weight 2 h before being sacrificed. Target skeletal tissues were harvested, fixed overnight at 4°C in 4% paraformaldehyde solution, and decalcified for two weeks in 0.5 M EDTA. Decalcified samples were embedded in paraffin and sectioned. BrdU-positive cells were detected using a BrdU-specific antibody (5-Bromo-2´-deoxy-uridine Labeling and Detection Kit I, Roche Diagnostic GmBH, Germany). The number of BrdU-positive nuclei was determined per one slice of growth plate specimen of the proximal tibia.

### Whole body analysis

Rats at seven and 33 weeks of age were subjected to whole body histological analysis. The rats were sacrificed by bleeding under deep anesthesia with isoflurane inhalation and the tissues were isolated and fixed in 10% neutral-buffered formalin solution. Tissues analyzed were as follows: cerebrum, cerebellum, pituitary, spinal cord, sciatic nerve, eye, optic nerve, harderian gland, auricle, tongue, submaxillary gland, esophagus, thyroid, parathyroid, thymus, heart, lung, thoracic aorta, spleen, liver, kidney, adrenal gland, pancreas, stomach, duodenum, ileum, colon, mesenteric lymph node, sternum, femur, bone marrow of the sternum and femur, skeletal muscle, skin, bladder, testis, epididymis, prostate, seminal vesicle, mammary gland, ovary, uterus, and vagina. Paraffin sections of each tissue were stained with H&E, and two pathologists histologically evaluated the slice sections by light microscopy independently, followed by review with the other pathologist.

### Statistical analysis

Statistical analysis of the data was performed using either a Student’s *t*-test or a two-way factorial analysis of variance (ANOVA), followed by a Turkey-Kramer test as a post hoc test. Data were expressed as means ± SE. *P* values less than 0.05 were considered statistically significant.

## Results

### Generation of CNP KO rats

**Fig 2:**
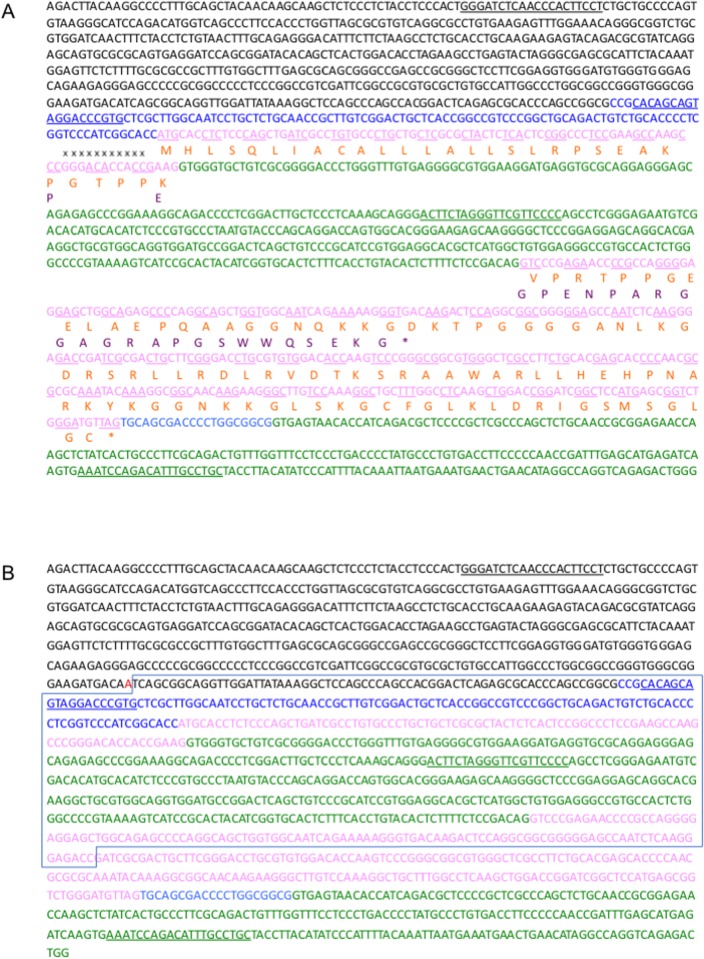
Sequence of the coding region of the rat CNP gene (*Nppc*) and its Δ11 mutation (A) and Δ774 mutation (B). (A) Blue, untranslated region; pink, coding region; green, intron. Amino acid sequence of WT CNP is aligned under the corresponding nucleotide sequence and depicted in orange. Eleven nucleotides marked with × (GGGACACCACC) were deleted in the Δ11 mutation. The deduced amino acid sequence after a mutation is aligned in purple. *, stop codon. Note that a premature stop codon occurred in the Δ11 mutant product. Four primer sequences listed in Table 1 were underlined. (B) Descriptions of the *Nppc* gene are the same as for the caption of (A). The boxed sequence is deleted in the Δ774 mutation and substituted by a single adenine (depicted in red).

**Fig 3:**
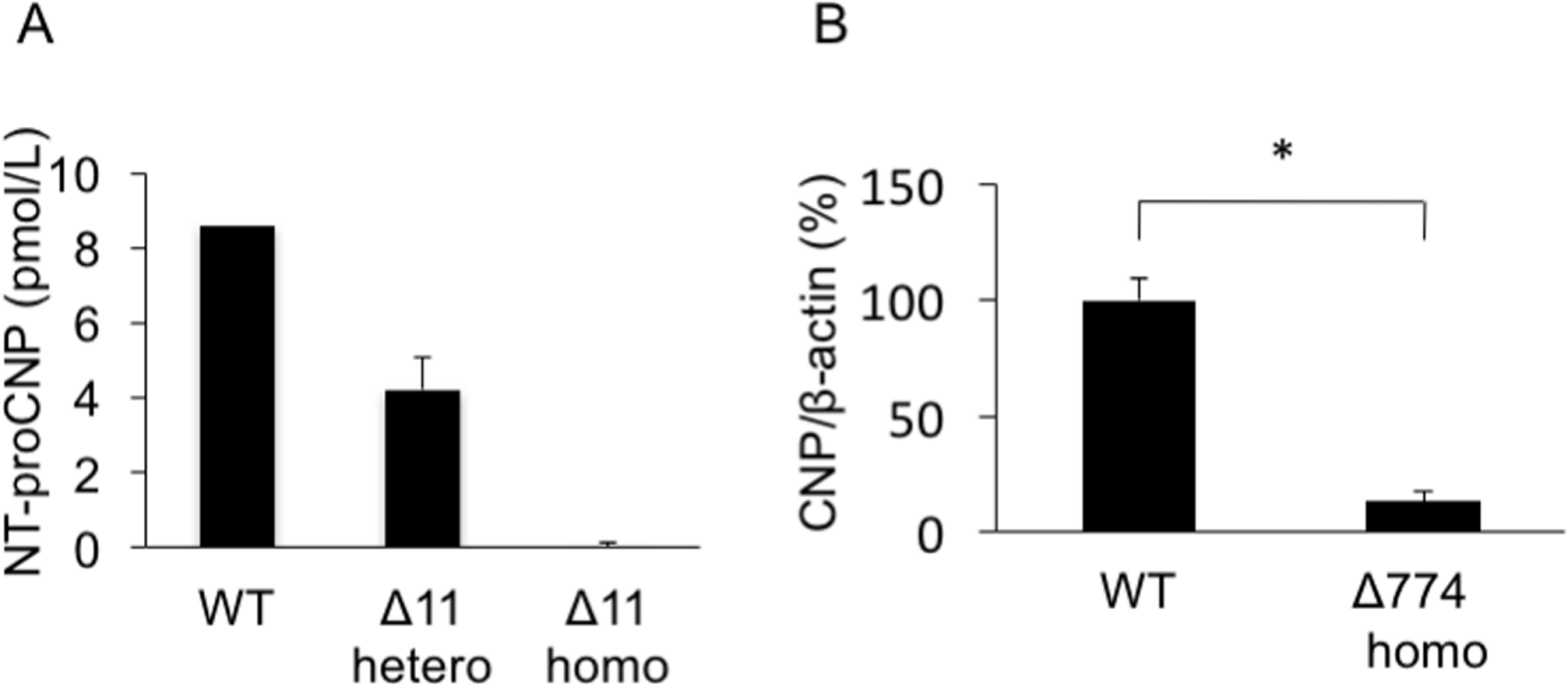
NT-proCNP levels and CNP mRNA expression levels in mutant rats. (A) Plasma NT-proCNP levels in Δ11 mutant rats. n = 2, 3, and 3 for female WT, heterozygous Δ11, and homozygous Δ11 rats, respectively. (B) CNP mRNA expression levels in the brains of Δ774 mutant rats, compared by those of WT rats as 100%. n = 3, each, *, *P* < 0.001.

In rats, as well as in humans and mice, CNP protein is initially synthesized as a 126-amino-acid preproCNP. After a signal peptide of 23 amino acids (a.a.) is released by cleavage, the residual proCNP is divided into two parts: the bio-inactive N-terminal (NT) portion (NT-proCNP) and the biologically active CNP of either 53 or 22 a.a. long (Fig 1A). By using ZFN-mediated genome-editing technology, we targeted a short portion (6 nucleotides) of exon 1 of rat *Nppc*, which is located near the N-terminal portion of proCNP (Fig 1A and 1B). We generated the objective ZFN mRNA and microinjected it into rat pronuclear stage zygotes that were subsequently transferred into recipients. In this process, we generated several viable offspring. We performed PCR-based screening and sequencing of tail biopsy DNA from the resulting offspring and confirmed that some of the founders possessed either monoallelic or biallelic mutations at the targeted *Nppc* locus (Fig 1C and Table 1). By crossing them with WT rats, we isolated four rat lines that were each heterozygous for a mutation in the *Nppc* gene encompassing deletions of 6, 9, 11, and 774-bp and were named Δ6, Δ9, Δ11, and Δ774, respectively. Among these four mutant lines, Δ6 was deduced to generate a two a.a. deletion (a.a. 28 and 29, Pro and Pro, NP_446202, at nucleotides 198–203, NM_053750.1), while Δ9 should generate a one amino-acid substitution (a.a. 26, from Gly to Ala, NP_446202, at nucleotides 192–194, NM_053750.1) and a three amino-acid deletion (a.a. 27–29, Thr, Pro and Pro, NP_446202, at nucleotides 193–201, NM_053750.1), within the N-terminal portion of the full-length CNP. By contrast, Δ11 (at nucleotides 192–202, NM_053750.1) generated a frame shift and a premature stop codon (at nucleotides 275–277, NM_053750.1) (Fig 2A). As for Δ774, this mutation was a massive deletion within the *Npcc* gene that included the translation initiation site (Fig 2B). In rats heterozygous for the Δ11 mutation, the plasma NT-proCNP level, which serves as a marker for CNP production, was only about half that in WT rats (WT, 8.45 and 8.75 pmol/L, n = 2, average, 8.60 pmol/L: Δ11 hetero, 4.21 ± 0.87 pmol/L, n = 3) and was not detectable in rats homozygous for the Δ11 mutation (−0.05 ± 0.16 pmol/L, n = 3) (Fig 3A). Quantitative RT-PCR revealed that the expression of CNP mRNA in the brain was drastically decreased in homozygous Δ774 mutant rats (Fig 3B).

In the Δ11 mutant rat line, 132 pups were born in total, and among them, homozygous KO, heterozygous KO and WT pups were 32 (24.2%), 79 (59.6%) and 21 (15.9%), respectively, which indicated that the CNP KO rats were born according to Hardy-Weinberg equilibrium and there would be no lethality in fetuses of CNP KO rats.

### Gross appearances and growth curves of CNP KO rats

**Fig 4:**
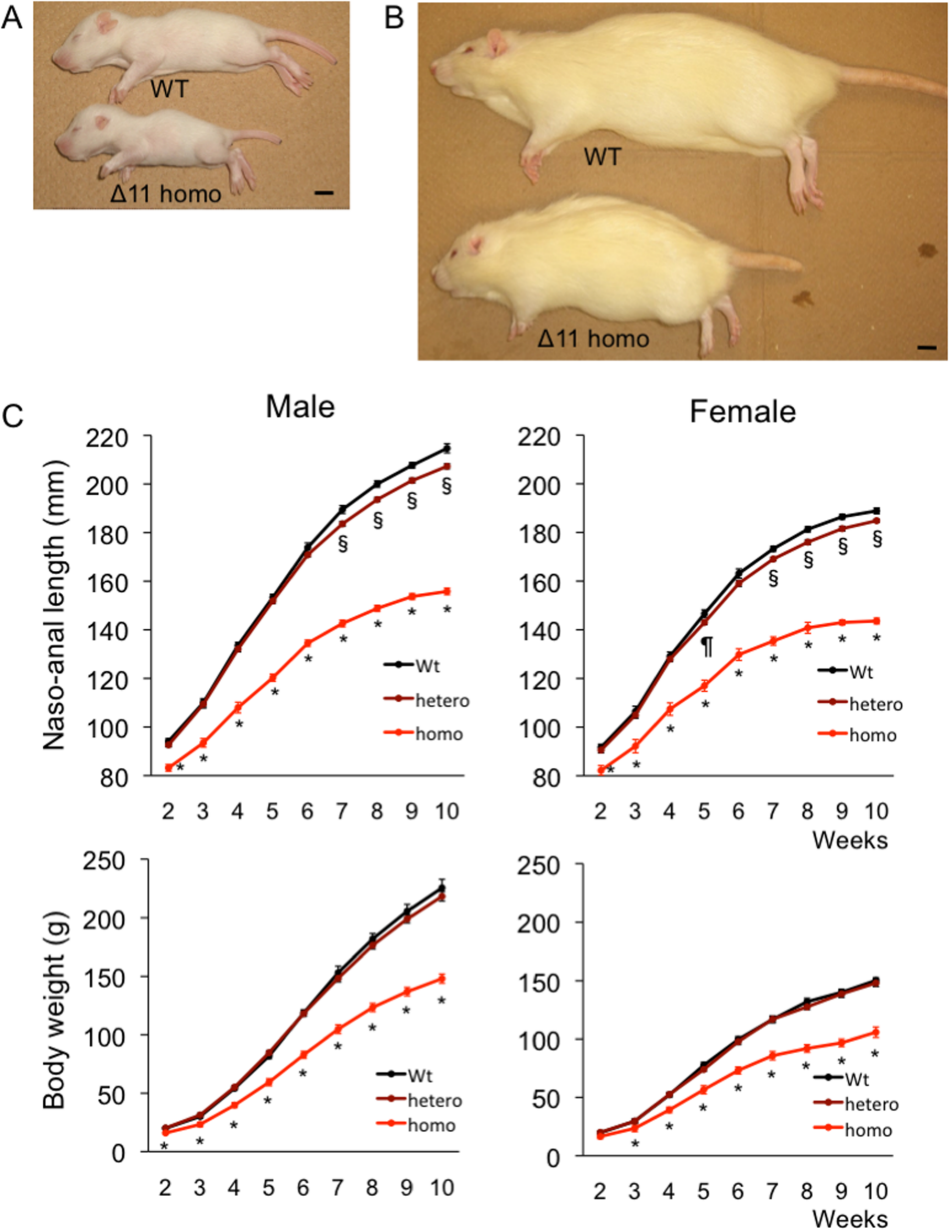
Gross appearance of and growth curves for Δ11 mutant rats. (A) and (B) Gross appearance of male WT rat and rat with a homozygous Δ11 mutation at two weeks (A) and 14 weeks (B) of age. Black scale bar in each picture denotes 10 mm. (C) Growth curves for Δ11 mutant rats. Upper panels show naso-anal lengths of male (left) and female (right) rats and lower panels exhibit body weight. n = 6, 15, and 10 for male WT, heterozygous Δ11, and homozygous Δ11 rats, respectively; n = 8, 14, and 5 for female WT, heterozygous Δ11, and homozygous Δ11 rats, respectively. *, *P* < 0.01 vs WT, §, *P* < 0.01 vs WT, and ¶, *P* < 0.05 vs WT.

**Fig 5:**
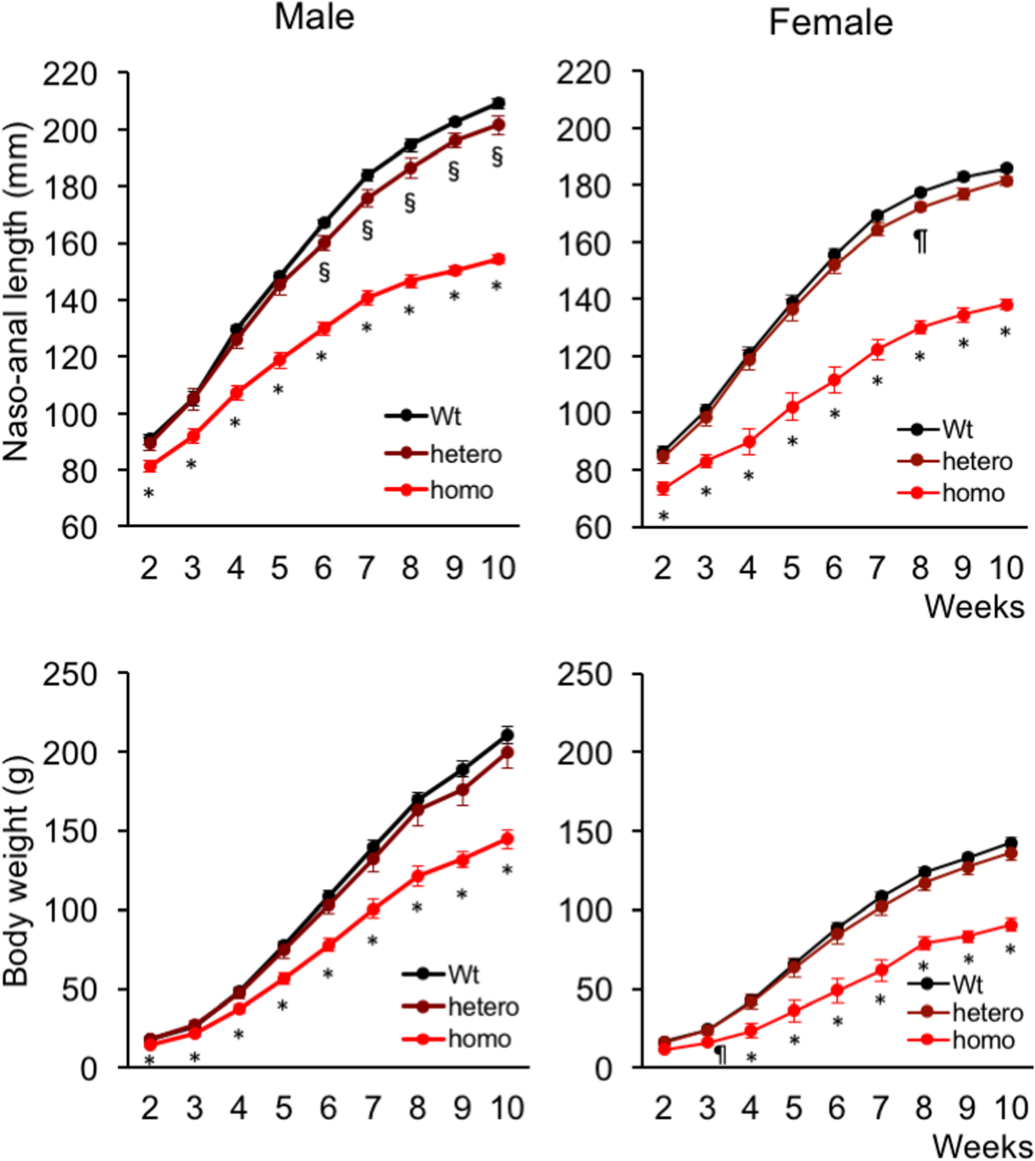
Growth curves for Δ774 mutant rats. Upper panels show naso-anal lengths of male (left) and female (right) rats and lower panels exhibit body weight. n = 6, 6, and 7 for male WT, heterozygous Δ774, and homozygous Δ774 rats, respectively; n = 11, 7, and 4 for female WT, heterozygous Δ774, and homozygous Δ774 rats, respectively. *, *P* < 0.01 vs WT, §, *P* < 0.01 vs WT, and ¶, *P* < 0.05 vs WT.

We subsequently intercrossed each line of heterozygous mutant rats and generated homozygous mutant rats. Homozygous Δ6 or Δ9 mutant rats showed a normal gross appearance. The growth curves based on naso-anal lengths of WT rats, rats heterozygous for either the Δ6 or Δ9 mutation, and rats homozygous for either the Δ6 or Δ9 mutation were identical. There were also no differences in the body weight of WT rats, rats heterozygous for either the Δ6 or Δ9 mutation, and those homozygous for either the Δ6 or Δ9 mutation. However, homozygous Δ11 mutant rats were obviously smaller than WT rats (Fig 4A and 4B). The growth curves show that homozygous Δ11 mutant rats were significantly smaller than WT rats at two weeks of age (Fig 4A and 4C). The difference later became more prominent and by 10 weeks of age, the naso-anal lengths of homozygous mutant rats were 72.6% and 76.0% of those of WT male and female rats, respectively (Fig 4C, upper panels). Although the naso-anal lengths of rats heterozygous for the Δ11 mutation did not differ from those of WT rats at earlier stages of growth, the naso-anal lengths of heterozygous Δ11 mutant male and female rats were significantly lower than those of WT rats after seven weeks of age (Fig 4C, upper panels). The body weights of homozygous Δ11 mutant rats were significantly lower than those of WT rats throughout the observation period except for two-week-old female rats (Fig 4C, lower panels). The weights of heterozygous Δ11 mutant rats did not differ from those of WT rats (Fig 4C, lower panels). Rats either homozygous or heterozygous for the Δ774 mutation exhibited the identical growth pattern as rats either homozygous or heterozygous for the Δ11 mutation, respectively (Fig 5). The naso-anal lengths of homozygous Δ774 mutant rats were 73.8% and 76.0% of WT male and female rats, respectively. The body weights of homozygous Δ774 mutant rats were significantly lower than those of WT rats during the observation period while those of heterozygous Δ774 mutant rats did not differ from WT rats, which is very similar to the respective Δ11 mutants. Based on these findings, we regarded homozygous Δ11 mutant rats and Δ774 mutant rats as CNP KO rats.

### Skeletal phenotypes of CNP KO rats

**Fig 6:**
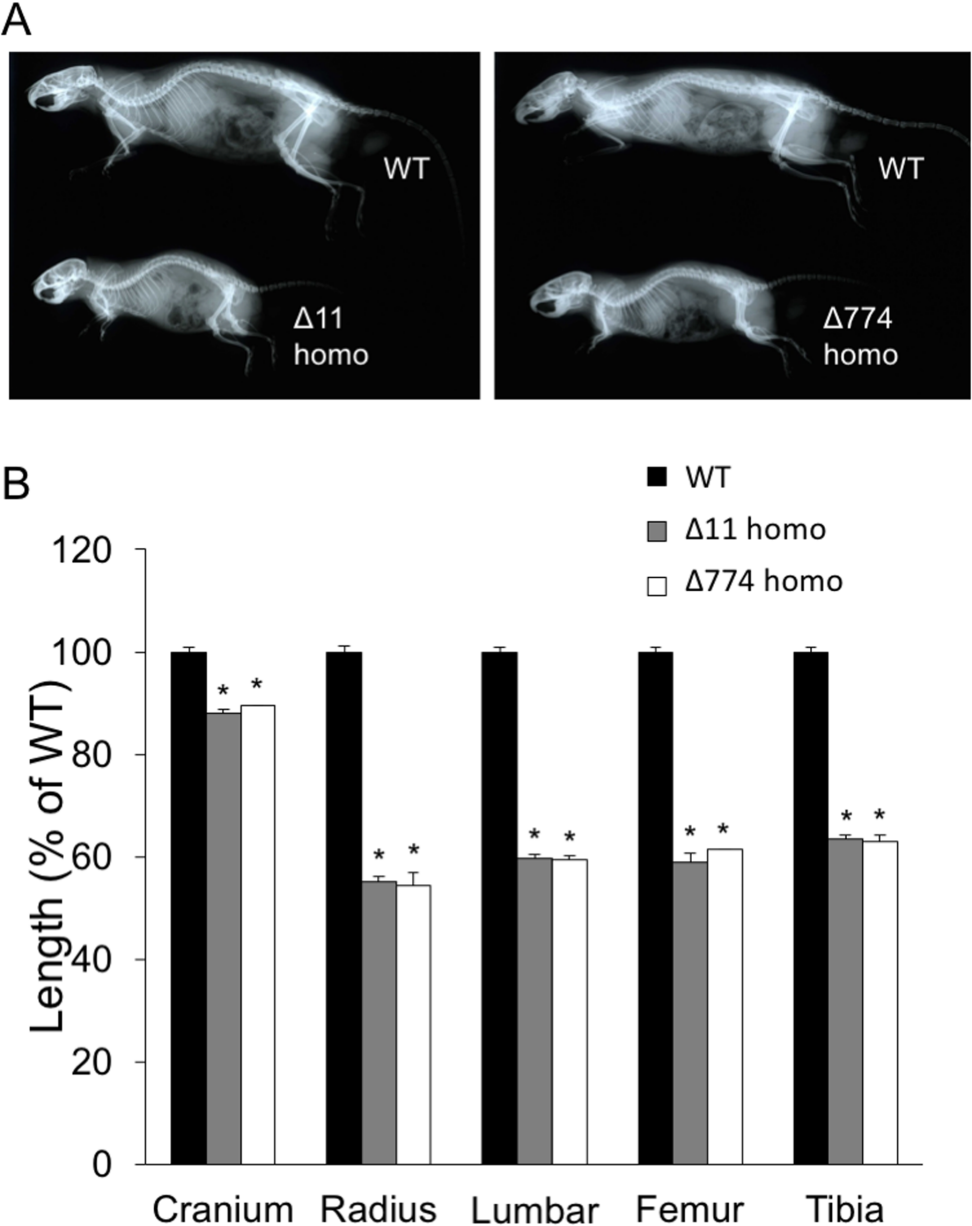
Skeletal analyses of CNP KO rats. (A) Soft X-ray pictures of homozygous Δ11 mutant (left) and homozygous Δ774 mutant (right) rats compared with WT rats. (B) Lengths of each indicated bone in male WT, homozygous Δ11, or homozygous Δ774 mutant rats at 20 weeks of age, measured from soft X-ray pictures. WT rats from both Δ11 and Δ774 lines were used for this analysis. n = 3–4, each, *, *P* < 0.01 vs WT.

Skeletal phenotypes of CNP KO rats at 20 weeks of age were analyzed on soft X-ray pictures. Both Δ11 and Δ774 homozygous mutant rats were shorter than WT rats to a similar extent due to impaired skeletal growth (Fig 6A). In both mutant rat lines, each bone formed through endochondral ossification was significantly shorter than in WT rats. The longitudinal length of the cranium was about 90% of that of WT rats, and the lengths of the radius, lumbar spine, femur, and tibia in Δ11 or Δ774 homozygous mutant rats were about 60% of those in WT rats (Fig 6B).

We also measured the bone mineral density of whole femurs of 8-week-old female rats, and found that there was no significant difference between CNP KO and WT rats (630.7 ± 8.6 and 637.1 ± 9.5 mg/cm^3^, n = 3 and 6, respectively).

### Histological analysis of the growth plates of CNP KO rats

**Fig 7:**
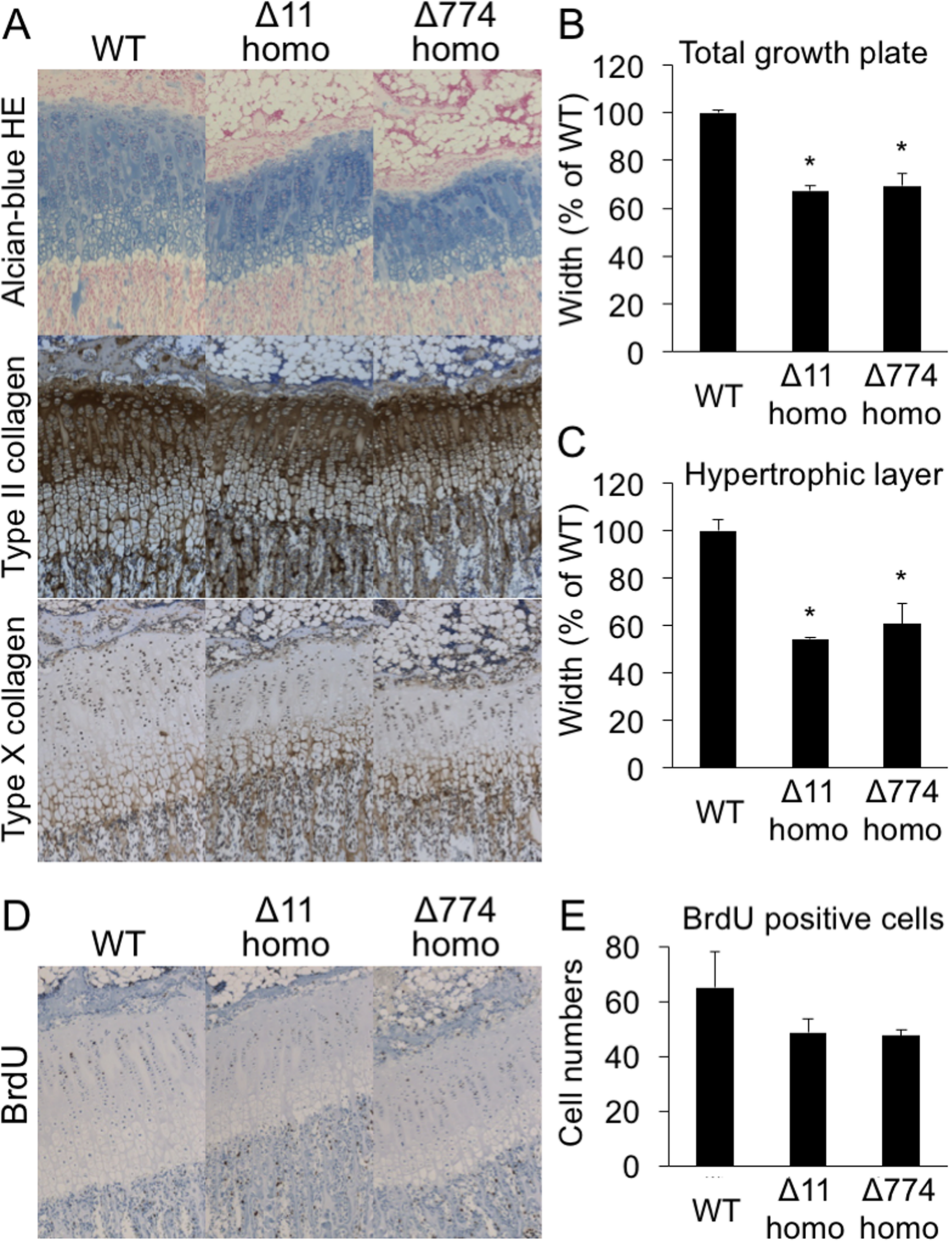
Histological analysis of the growth plates of CNP KO rats. (A) Histological pictures of tibial growth plates of three-week-old male WT, homozygous Δ11, and homozygous Δ774 mutant rats. WT rats from Δ774 mutant line were used for this analysis. Upper panels show the results of Alcian blue-H&E staining, while the middle and lower panels show the results of immunohistochemical staining for type II and type X collagens, respectively. (B) and (C) Graphs of the widths of total growth plates (B) and their hypertrophic chondrocyte layers (C). n = 3 in each group and each value is expressed as a ratio (%) of the WT value. *, *P* < 0.01 vs WT. (D) Histological pictures showing BrdU staining of the tibial growth plates of three-week-old male WT, homozygous Δ11, and homozygous Δ774 mutant rats. (E) Graphs of the number of BrdU positive chondrocytes in the proliferative chondrocyte layers of the growth plates.

Next, we performed histological analysis of the tibial growth plates of three-week-old CNP KO rats. As shown in Fig 7A and 7B, the width of the growth plates in the Δ11 and Δ774 homozygous mutant rats was drastically reduced compared with that in WT rats. Among the layers of the growth plate, the hypertrophic chondrocyte layer, which is detected by immunostaining for type X collagen (Fig 7A, lower panels), was the most severely affected in CNP KO rats (Fig 7A and 7C). Analysis of BrdU staining of the growth plates showed that chondrocytes positive for the immunohistochemical BrdU stain in proliferative chondrocyte layers tended to be less numerous in CNP KO rats compared with WT rats (Fig 7D and 7E).

### Mortality of CNP KO rats

We have previously reported that the survival rate of systemic CNP KO mice is about 40% with solid food and 70% with pulverized food at 10 weeks of age [7]. We determined the mortality of CNP KO rats and found that the mortality of Δ11 (n = 25) or Δ774 (n = 11) mutant rats was the same as that of WT rats (n = 15 and 16, respectively) and remained at 0% at 10 weeks of age. We also confirmed that CNP KO rats were alive over one year after birth.

### CNP KO rats at 33 weeks of age

**Fig 8:**
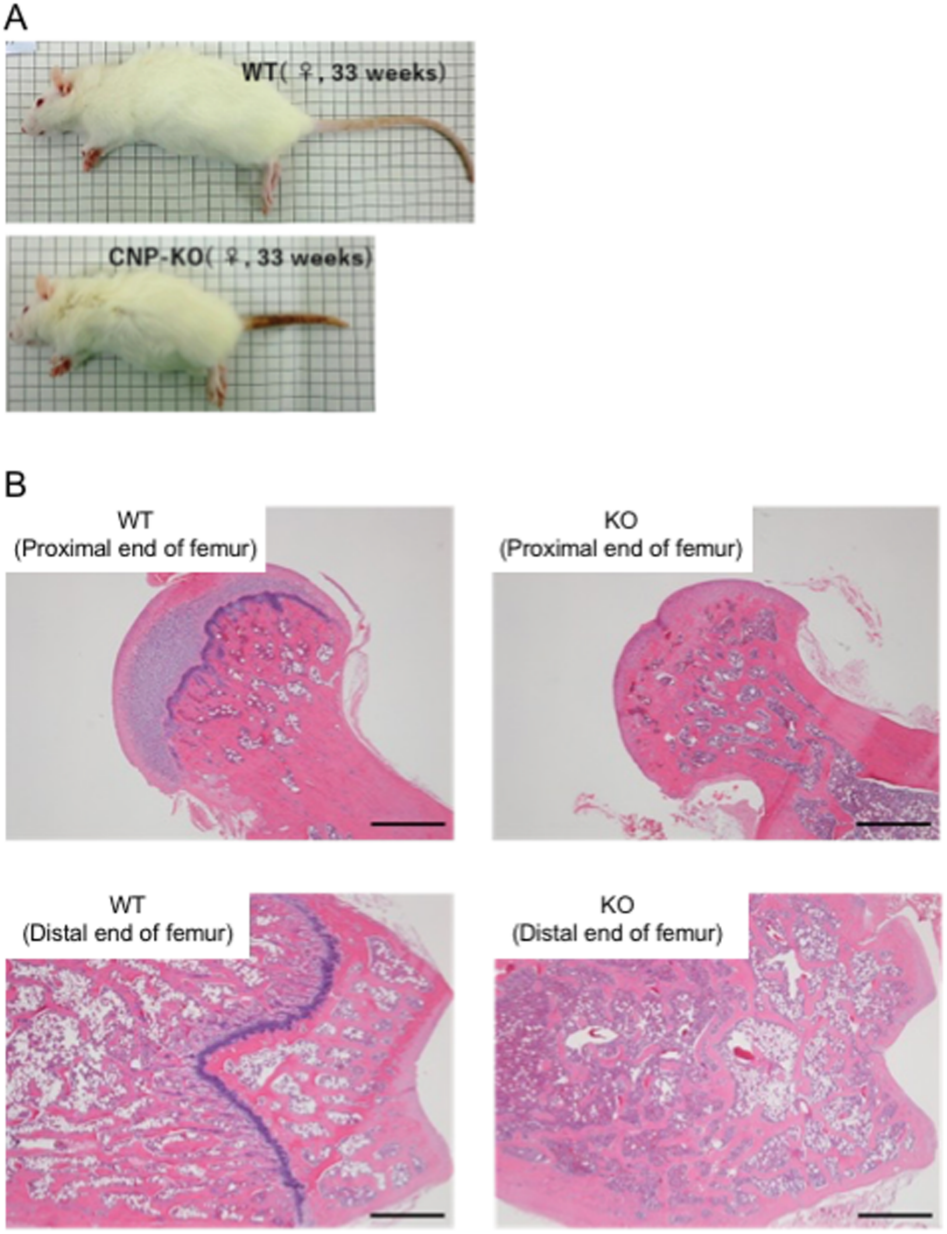
Gross appearance and femoral growth plates of CNP KO rats at 33 weeks of age. (A) Gross appearance of female WT (left) and homozygous Δ11 mutant (right) rats. (B) Typical examples of an H&E-stained specimen of the femoral bone (proximal and distal region) in WT and KO rats at 33 weeks of age. Scale bar shows 1 mm.

Since Δ11 and Δ774 lines of CNP KO rats showed almost identical phenotypes, we used homozygous Δ11 rats and WT rats for subsequent analyses. As described above, CNP KO rats do not have short lives, and no death was observed by the age of 33 weeks. Therefore, we analyzed phenotypes of female CNP KO rats at 33 weeks of age and compared with those of female WT rats. Body length and body weight of CNP KO rats at 33 weeks of age were significantly lower, specifically 72.2% and 62.1% of WT rats (Fig 8A and Table 2). The heart and kidney weights were also significantly lower in CNP KO rats than those in WT rats (Table 2), possibly due to marked less body length and body weight of CNP KO rats.

**Table 2:** Body length, body weight, heart weight, and kidney weight of female CNP KO and WT rats at 33 weeks of age.

Genotype	CNP KO	WT
n	3	3
Body Length (mm)	151 ± 2**	209 ± 2
Body Weight (g)	132.8 ± 1.4**	213.7 ± 7
Heart Weight (g)	0.47 ± 0.005**	0.634 ± 0.029
Kidney Weight (g)	1.12 ± 0.016**	1.408 ± 0.051

Data represent the means ± SE of CNP KO (homozygous Δ11 mutant) and WT rats. Bilateral kidney weights are shown.

**, *P* < 0.01 vs WT rats.

We also analyzed the growth plates at proximal and distal ends of femurs of rats at 33 weeks of age. Fig 8B shows typical examples of growth plate specimens in the proximal and distal ends of femurs. The femoral growth plates in all CNP KO rats were closed, while they were still observed in all WT rats (S1 Table).

### Whole body histology of CNP KO rats

**Fig 9:**
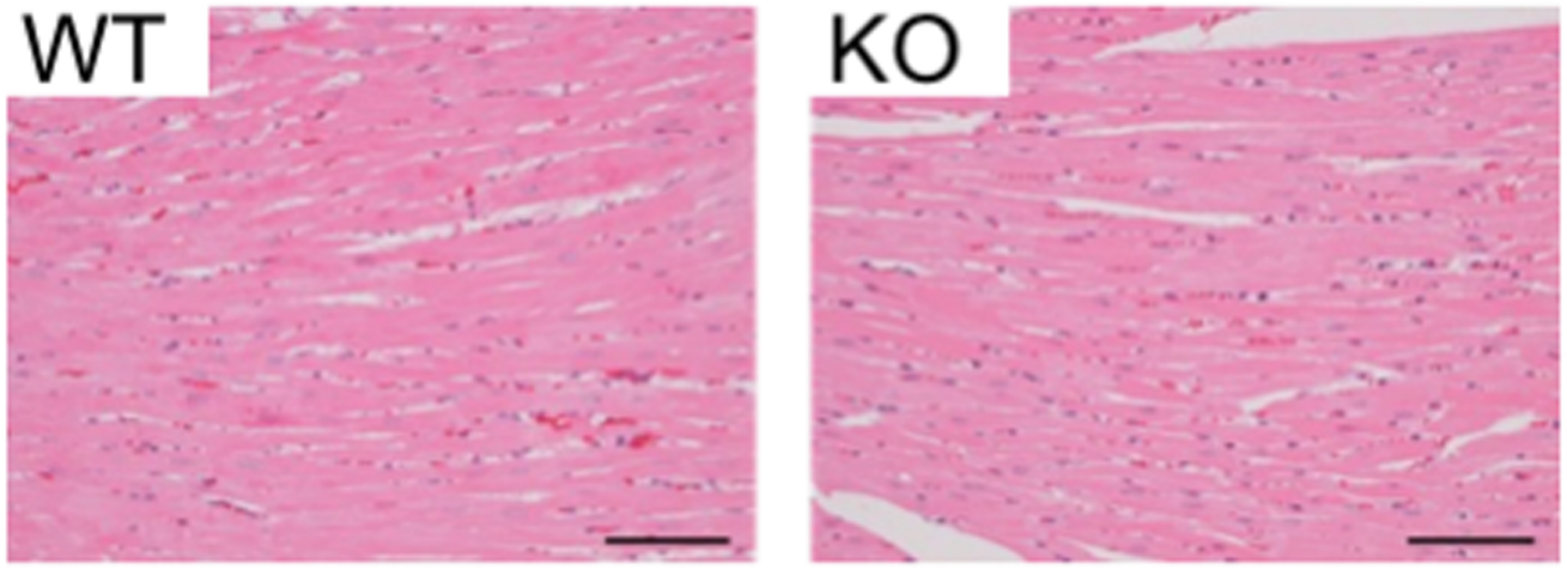
Typical examples of H&E-stained specimens of the heart of female WT (left) and CNP KO (homozygous Δ11 mutant, right) rats. Scale bar shows 1 mm.

We histologically analyzed whole body tissues of CNP KO and WT rats at seven and 33 weeks of age. Several histological lesions were observed in both CNP KO and WT rats, but there were no differences in the severity or frequency of these changes between CNP KO and WT rats at both ages: these lesions were considered to be accidental or age-related (S2 Table). As described above, the heart and kidney weights were significantly less in CNP KO rats than in WT rats at 33 weeks of age (Table 2), but none of the CNP KO and WT rats showed severe pathological lesions, such as cardiac hypertrophy, necrosis, or fibrosis (Fig 9 and S2 Table).

## Discussion

We have recently revealed that the CNP/NPR-B system is a potent stimulator of endochondral bone growth using genetically targeted mice [3–11]. However, both systemic and cartilage-specific CNP KO mice died early [3, 7], and we could not sufficiently analyze the effect of CNP deficiency at the adult age. In the present study, we succeeded in the generation of CNP KO rats using ZFN technology. CNP KO rats showed impaired skeletal growth, and their long bones were shorter and the growth plates were narrower than those of WT rats. These results agree well with the findings in CNP KO mice. The novel findings for CNP KO rats were that they lived for over one year and CNP deficiency histologically affected only the bones among all body tissues studied.

Rat models are often said to be more suitable for understanding human physiology than mouse models [26]. Therefore, we generated rats deficient in the CNP-encoding gene and analyzed their phenotypes. As for the methods to generate gene mutations in rats, several methods are available, including transposon-mediated [30] and N-ethyl-N-nitrosourea [31, 32] mutagenesis. Furthermore, recent progress in newly developed genome editing technologies has enabled us to generate more effectively targeted gene disruptions in rats. These new developments include ZFN technology [27], transcription activator-like effector nuclease (TALEN) technology [33], and the clustered, regularly interspaced, short palindromic repeats (CRISPR)-associated (Cas) protein system [34]. In this study, we utilized ZFN-mediated genome editing technology to generate CNP KO rats. Ours is the first report concerning the generation and characterization of CNP KO rats. Out of the four mutant rat lines that we generated, the Δ11 mutation causes a frame shift with a premature stop codon in the *Nppc* gene and the Δ774 mutation encompasses a large deletion within *Nppc* that includes the translation initiation site. These mutations should result in null alleles of the *Nppc* gene. NT-proCNP, a marker for CNP expression in the plasma, and the CNP transcript in the brain were not detected in homozygous Δ11 and Δ774 mutant rats, respectively. The skeletal phenotype of Δ11 mutant rats was identical to that of Δ774 mutant rats, suggesting that the observed skeletal phenotype is due to null mutations in the rat CNP gene.

With regard to the skeletal phenotypes of CNP KO rats, the quality and quantity of impaired endochondral bone growth were the same as those observed in CNP KO mouse models [3–7]. A small but significant decrease in the length of heterozygous CNP mutant rats was also observed in mice heterozygous for a spontaneous loss-of-function mutation in the *Nppc* gene (long bone abnormality: lbab) [6]. Interestingly, Hisado-Oliva et al. recently reported two cases of impaired skeletal growth caused by heterozygous *NPPC* mutations, one of which corresponded to that observed in lbab mice [19]. Because heterozygous mutations in the NPR-B as well as CNP genes increasingly attract attentions as the cause for short stature and impaired skeletal growth [14–19], our heterozygous CNP mutant rat would be a valuable model for these conditions, especially for those with *NPPC* mutations. Nevertheless, it is not unclear at present why the difference in length of heterozygous CNP mutant rats from WT rats is small in spite of half of CNP levels. Any local regulations including those of expression of NPR-B or NPR-C, the clearance receptor for CNP, may contribute. We are now planning to administer CNP ligand to CNP KO rats and estimate the dosage response of CNP in skeletal growth and investigate the plausible mechanism of this phenomenon.

The body weight of CNP KO rats was observed to be lower than that of WT rats, as has previously been found for CNP KO mice [3, 7]. We have not elucidated the exact mechanism, but we believe that impaired skeletal growth somehow affects body weight because cartilage-specific CNP KO mice with impaired skeletal growth exhibit decreased body weight, just like systemic CNP KO mouse models [7].

One of the prominent findings of the present study is that the survival rate of CNP KO rats is identical to that of WT rats, in contrast to CNP KO mice, whose survival rate drops to less than 70% at 10 weeks of age [7]. As for the cause of increased mortality by CNP depletion, we speculated that the impaired skeletal growth is responsible because the mortality of cartilage-specific CNP KO mice is as high as that of systemic CNP KO mice. Especially, we hypothesized that the severe malocclusion sometimes observed in CNP KO mice prevents the consumption of solid foods and relates to their early death [7]. In accordance with this idea, all CNP KO rats we examined had no malocclusions and did survive for over one year. The impaired endochondral bone growth was similarly observed in both CNP KO mice and KO rats, and the reason for no malocclusion in CNP KO rats is not well understood at this time. This finding also demonstrates that although CNP is a universal and crucial stimulator of physiological endochondral bone growth in mammals, CNP is not necessary for survival in at least one mammalian species.

Survival of CNP KO rats over one year enabled us to examine the skeletal and whole body over a longer term. When we evaluated body length and growth plates at 33 weeks of age, the body lengths of CNP KO rats were significantly shorter, around 72% of WT rats, which was similar with that at 10 weeks of age. These results indicated that the final height was also lower in CNP KO rats. Interestingly, the growth plates of the femur were closed in all CNP KO rats but not in WT rats at 33 weeks of age, indicating that growth plates closed early in CNP KO rats. The narrowing of the growth plate in CNP KO rats might result in the early closure of the growth plate, but the exact mechanism is still unclear. Moreover, the mechanism of growth plate closure itself is not completely understood yet in rodents as well as in humans [35]. On the other hand, the mechanism of growth plate closure would have clinical relevance, because precocious puberty is a known cause of short stature caused by premature closure of growth plates in long bones due to earlier puberty [35]. Conversely, CNP KO rats might be valuable animal models for investigating the mechanism of growth plate closure and developing compounds that affect growth plate closure. Further studies including on the time course of the growth plate closure in CNP KO rats will be necessary.

Previously, we reported that CNP is a pivotal stimulator of endochondral bone growth in the craniofacial region and plays an important role in midfacial skeletogenesis using CNP overexpression and CNP KO mice [36]. We are now analyzing the craniofacial region, including the foramen magnum, of CNP KO rats to clarify the effect of CNP deficiency on facial hypoplasia and spinal canal stenosis, which are known to be complications associated with achondroplasia [37, 38].

As described above, the most obvious phenotypes observed in rats or mice with systemic deficiency in CNP is impaired skeletal growth. Since CNP and NPR-B are ubiquitously expressed in the body, e.g., in the brain, cardiovascular system and gonads [1, 2], the extra-skeletal defects could be expected in CNP KO rats. However, other than the bones, we did not observe any abnormality in all tissues studied in CNP KO rats, at least histologically. Langenickel et al. reported that transgenic rats expressing a dominant-negative mutant of NPR-B exhibited cardiac hypertrophy at 6 months old [39]. In contrast, the heart weight of CNP-KO rats was significantly lower compared with that of WT rats, and we did not observe cardiac hypertrophy in CNP KO rats. We do not exactly know the reason for the discrepancy between our data and their data. Since NPR-B is known to have constitutive activity and cartilage specific-NPR-B KO mice showed more severe skeletal phenotypes than did cartilage-specific CNP KO mice [7], the deficiency in the ligand CNP in our study might result in the different cardiac phenotypes in NPR-B deficient rats. Considering that ANP KO mice showed an over 40% increase in heart weight to body weight ratio [40], it is plausible that CNP deficiency does not greatly affect cardiac hypertrophy. Regarding gonadal function, our female CNP KO rats were infertile as were the female NPR-B KO mice [4], but we did not find any abnormality in the ovary and uterus of female CNP KO rats. Further functional studies will be necessary for the elucidation of the mechanism of infertility in female CNP KO rats. CNP KO rats would be useful to analyze the physiological function of CNP in gonads and the cardiovascular and central nervous systems because of their larger size than mice, which permits manipulations difficult to perform with mice. One such example is the measurement of blood pressure and heart rate.

Considering human disease relevant to CNP KO rats, we must compare the phenotypes of CNP KO rats with those of acromesomelic dysplasia, type Maroteaux (AMDM), which is caused by biallelic mutations in the gene encoding NPR-B, the bioactive receptor for CNP [12]. In accordance with our previous reports showing that the impaired skeletal growth phenotypes in CNP KO mice and NPR-B KO mice are qualitatively identical [3, 4, 7], the written skeletal phenotypes observed in AMDM are fundamentally equal to those seen in CNP KO rats [12, 13, 16]. However, explicit descriptions on early growth plate closure, which is one of the most striking skeletal phenotypes observed in the CNP KO rats, do not exist in previous reports on AMDM patients. Further researches on early growth plate closure in AMDM patients, probably by using x-ray pictures, would be necessary. On the other hand, there are no reports of early death of AMDM patients: as for longevity, the CNP KO rat model might be more similar to AMDM patients than mouse models with CNP or NPR-B deficiency.

In conclusion, we generated rats deficient in CNP using ZFN-mediated genome editing technology. These rats exhibited impaired endochondral bone growth, as do CNP KO mice, but they were not short-lived. Histologically, there was no abnormality in tissues other than bones in CNP KO rats. CNP KO rat is a good animal model for the analysis of the effects of CNP/NPR-B signaling on endochondral bone growth. Furthermore, as an excellent model for impaired skeletal disorder, CNP KO rats would be suitable for the evaluation of the therapeutic effects of drug candidates for growth disorder through the functional and morphological analyses of skeletal tissues. These rats would also be beneficial for the evaluation of the physiological functions of CNP in extra-skeletal tissues.

## Supporting information

S1 Table Histological observations of femoral growth plates in CNP KO and WT rats at 33 weeks of age. CNP KO rats shown in this table were homozygous Δ11 mutants.
(XLSX)

S2 Table Summary of histological findings of each rat. Grade of findings: normal (0), slight (1), moderate (2), severe (3). CNP KO rats shown in this table were homozygous Δ11 mutants. The rats at 33 weeks of age were the same individuals in S1 Table. The other organs/tissues not listed in this table were normal in all rats except for femur [S1 Table].
(XLS)
